# Sleep-driven prefrontal cortex coordinates temporal action and multimodal integration

**DOI:** 10.1186/s13041-025-01175-0

**Published:** 2025-01-23

**Authors:** Ahmed Z. Ibrahim, Kareem Abdou, Masanori Nomoto, Kaori Yamada-Nomoto, Reiko Okubo-Suzuki, Kaoru Inokuchi

**Affiliations:** 1 Research Centre for Idling Brain Science, University of Toyama, Toyama, 930-0194 Japan; 2 Department of Biochemistry, Graduate School of Medicine and Pharmaceutical Sciences, University of Toyama, Toyama, Japan; 3 CREST, Japan Science and Technology Agency (JST), University of Toyama, Toyama, Japan; 4 Department of Biochemistry, Faculty of Pharmacy, Cairo University, Cairo, 11562 Egypt; 5 Present Address: College of Pharmacy, Al-Ain University, Abu Dhabi, UAE

**Keywords:** Cognitive flexibility, Idling, Sleep, Prefrontal cortex, Multi-modal integration, Temporal actions

## Abstract

Cognitive processes such as action planning and decision-making require the integration of multiple sensory modalities in response to temporal cues, yet the underlying mechanism is not fully understood. Sleep has a crucial role for memory consolidation and promoting cognitive flexibility. Our aim is to identify the role of sleep in integrating different modalities to enhance cognitive flexibility and temporal task execution while identifying the specific brain regions that mediate this process. We have designed “Auditory-Gated Patience-to-Action” Task in which mice should process different auditory signals before action execution as well as analyzing the visual inputs for feedback of their action. Mice could learn the task rule and apply it only after sleeping period and could keep the performance constant across sessions. c-fos positive cells showed the involvement of prelimbic cortex (PrL) during task execution. Chemo-genetic inhibition verified that PrL is required for proper signal response and action timing. These findings emphasize that sleep and cortical activity are keys for cognitive flexibility in adapting to different modalities.

**Supplementary Information:**

The online version contains supplementary material available at 10.1186/s13041-025-01175-0.

## Introduction

Cognitive flexibility is a crucial cognitive skill that allows individuals to shift their thinking and actions in response to new situation [1, 2]. While awake neural circuits contribute to cognitive flexibility, the processes occurring during sleep play a more crucial role [3, 4]. The consolidation of newly learned information is significantly enhanced during sleep through neural reactivation and synaptic plasticity [5, 6].

In our daily lives, we constantly rely on multiple senses to make decisions and take actions at the right time. For example, when we cross the street, we use the traffic lights as visual signals and sounds of approaching cars as auditory cues to determine the correct timing to go. However, how our brain integrates these different sensory inputs for temporal action execution is not fully understood.

It is widely known that the prefrontal cortex (PFC), due to its high-level connectivity within brain networks [7], contributes to a wide range of cognitive functions [3, 7, 8]. These functions include attention with integrating multiple modalities [8, 9], memory consolidation for decision-making processes [10] and time-based actions [11]. Many studies addressed memory acquisition and recall in spatial navigation tasks and working memory tasks for short-term maintenance of information [12, 13]. However, how sleep consolidates memories for learning a complex task and its long-term application that requires integration of different sensory inputs together with temporal precision are not fully understood. Our aim is to unveil the role of sleep in the learning and utilization of a flexible rule in multifaceted task and the brain region coordinating this process. In this study, we addressed the role of PFC activity and idling timepoints that aid in learning and applying multiple modalities task rule with temporal precision.

## Results

**Fig. 1:**
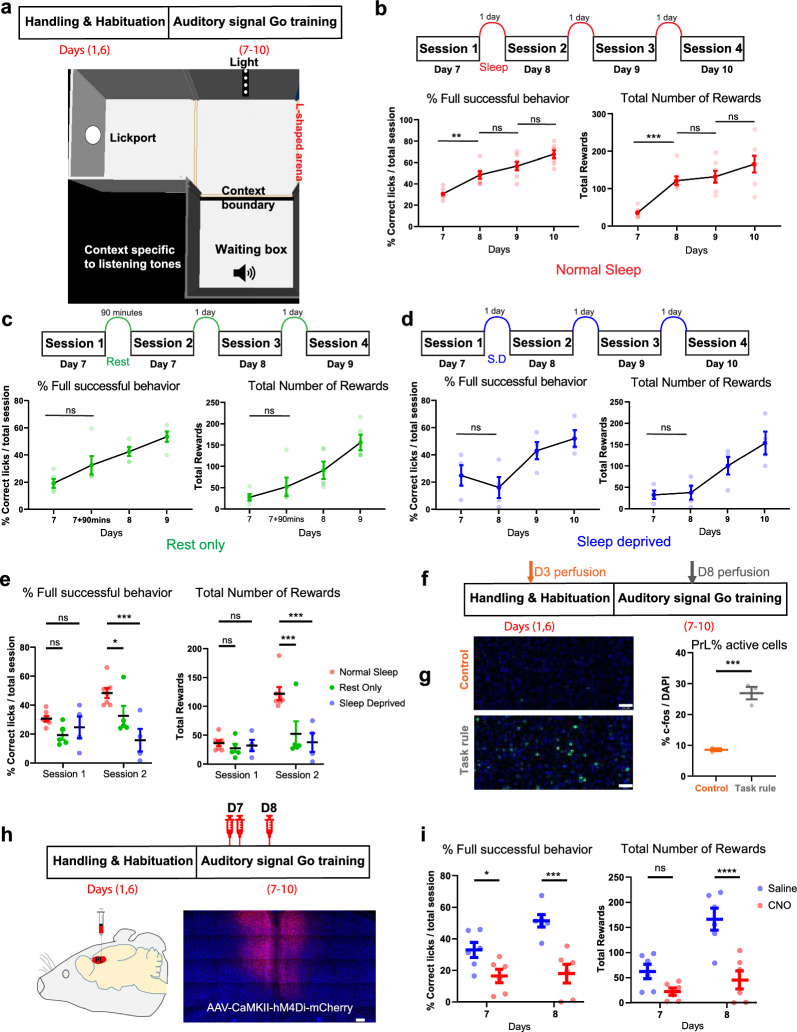
Prefrontal cortical activity and sleep are crucial for learning task structure. **a** *Top*: Behavior schedule for multiple modalities behavior paradigm. *Bottom*: Representative diagram for L-shaped arena and significance of different contexts. **b** *Top*: Behavior timeline for normal sleep group. *Bottom left*: Percentage of successful trials in calculated by percentage of rewards earned compared to total initiated trials. *Bottom right*: Number of rewards given (n = 7) (One-way RM ANOVA, Šidák`s multiple comparison). **c** *Top*: Behavior timeline for rest only group. *Bottom left*: Percentage of successful trials. *Bottom right*: Number of rewards given (n = 5) (One-way RM ANOVA, Šidák`s multiple comparison). **d** *Top* Behavior timeline for sleep deprived group. *Bottom left* Percentage of successful trials. *Bottom right* Number of rewards given (n = 4) (One-way RM ANOVA, Šidák`s multiple comparison). **e** *Left* Comparison between 3 groups at first 2 sessions in terms of percentage of successful trials. *Right* Comparison between 3 groups at first 2 sessions in terms of rewards earned (Two-way RM ANOVA, Šidák`s multiple comparison). **f** Behavior schedule with 2 highlighted timepoints for different groups (n = 3/group). **g** *Left*: Representative image of PrL at different timepoints. *Blue* (DAPI). *Green* c-fos stained cells. *Scale bar* 50 µm. *Right* Percentage of active cells in Prl between different groups (Ordinary One-way ANOVA, Šídák’s multiple comparison). **h** *Top*: Behavior schedule used to manipulate PrL during first 2 behavior sessions. *Bottom left*: Labeling of PrL with hM4Di-mCherry. *Right*: Representative image for PrL coronal section with native fluorescence of hM4Di-mCherry protein expression (*red*). *Blue* (DAPI). *Scale bar* 200 µm. **i** *Left*: Comparison between groups at first 2 sessions in terms of percentage of successful trials. *Right*: Comparison at first 2 sessions in terms of rewards earned (Two-way RM ANOVA, Šidák`s multiple comparison) (n = 6/group). *P < 0.05; **P < 0.01; ****P < 0.0001; ns, not significant (*P* > 0.05). Data are presented as the mean ± s.e.m

We developed a self-initiated behavioral paradigm in an L-shaped arena in which mice are required to integrate multiple sensory modalities successfully to obtain a sucrose reward, a behavior that we termed “Auditory-Gated Patience-to-Action Task” (Fig. 1a). In brief, mice are trained to hold their action and wait while presenting an initial cue followed by a hold cue; both cues are 6 kHz tone. After presenting a 12 kHz tone, mice would get the chance to act upon and approach the lick-port to get a sucrose reward. Besides giving attention to the auditory cues, mice can integrate the significance of light visual cues as feedback for their action timing. Mice could learn self-initiating the trials, waiting for tone signals, exiting context and getting a reward. Mice can start understanding the rule structure and comprehend task rules by the 2nd session on day 8. This could be identified by the significantly higher number of rewards acquired or the percentage of fully successful trials by going through all the required steps accurately compared to the 1st session on day 7 (Fig. 1b). Success of mice in learning the task rule might be attributed to several possibilities that include multiple sessions exposure (i), resting time (ii), incubation period (iii) or sleep (iv), which is crucial for multiple-modalities integration. To confirm these different possibilities, the same session was applied but on a different time schedule: rest only group by subjecting mice to session 2 on the same day 90 min later after session 1, Sleep deprived group that followed the same time schedule but were subjected to sleep deprivation after session 1. While rest only group showed a slight improvement, sleep deprived group showed a slight decline in performance. Nevertheless, both groups, rest only group (Fig. 1c) and sleep deprived group (Fig. 1d), did not show significant improvement in number of rewards or percentage of successful behavior in session 2 compared to session 1. Although all 3 groups showed non-significant difference on session 1, normal sleep group showed significantly higher number of rewards and higher timing precision in session 2 (Fig. 1e). Next, to investigate the brain region involved in the integration of multiple modalities and the resolution of temporal action execution, we used c-fos positive cells counting by immunohistochemistry for mice perfused 90 min after different behavioral sessions: (D3), control water restriction before any behavioral learning and (D8) after learning the task rule (Fig. 1f). Pre-limbic (PrL) cortex showed involvement after learning the task by showing higher number of active cells compared to control (Fig. 1g). To investigate the necessity of the PrL in the integration of sensory information and action planning, we manipulated PrL area chemogenetically using designer receptors exclusively activated by a designer drug (DREADD). Mice received bilateral injections of adeno-associated virus 2/9 (AAV2/9), encoding Ca2+/calmodulin-dependent protein kinase IIa (CaMKIIa)-hM4Di-mCherry, targeting the mPFC (PrL). PrL was silenced through administering the exogenous ligand clozapine n-oxide (CNO) on day 7 and 8 for task learning and during executing it (Fig. 1h). By inhibiting PrL neural dynamics, mice failed to learn the task structure on day 7 in task encoding and its application recall stage on day 8 manifested by showing a significantly lower number of reward acquisition and imprecise behavior execution (Fig. 1i). This indicates that PrL region plays an important role in integrating multimodal sensory cues for accurate time-directed decision making.

## Discussion

Sleep is known to support cognitive functions and creative problem-solving, with the PFC being a pivotal brain region in mediating these processes [3, 4, 6]. In our work, through a task involving multiple modalities that necessitates simultaneous sensory integration and time-precise actions, we highlighted the critical role of sleep and PrL activity in ensuring precise task execution. However, the causal relationship between cortical activity and sleep remains to be determined using optogenetics and calcium imaging [3]. Although in this behavior task, mice need to integrate multiple modalities for task execution, control experiment using a single modality would further confirm the multi-modal integration. Chemogenetics experiment showed the necessity of PrL for task execution, but other surrounding brain regions might be affected. Moreover, control mCherry group would rule out any effect caused by CNO injection. Effective decision-making relies on the capacity to attend to various sensory signals, integrate their relevance, and calculate the time necessary for action [14]. Given the extensive connectivity between the PFC and the hippocampus, which contains time cells for temporal coding [15], as well as the mediodorsal thalamus that sustains task rule representation in the PFC [8], this leaves the exact circuit underlying our task behavior yet to be determined. Deficits in cognitive flexibility are tied to various disorders, and uncovering the neural basis may aid in their treatment.

## Methods

### Animals

Naïve wild-type male C57BL/6J mice were purchased from Sankyo Labo Service Co. Inc. (Tokyo, Japan) and maintained on a 12 h light/dark cycle at a controlled temperature 24 ± 3 °C) and humidity (55 ± 5%) with free access to food and water. Mice used in all behavioral experiments were 12–18 weeks old. All behavior experiments were done during the early light cycle.

### Viral vectors

*For chemogenetics experiment*, the AAV viral vector AAV-CaMKIIa-hM4Di-mCherry (8.87 × 10^14^ vg/ml) was used. pAAV-CaMKIIa-hM4Di-mCherry was acquired from Addgene (Cambridge, MA, Plasmid #50477).

### Drugs

To prepare clozapine-n-oxide (CNO) (C0832, Sigma-Aldrich), 5 mg of CNO was dissolved in 500 µl of dimethyl sulfoxide (DMSO) (049-07213, FUJIFILM Wako Pure Chemical), and then the solution was diluted to a total volume of 10 ml with saline which was intraperitoneally (i.p.) injected at a dose of 5 mg/kg.

### Stereotactic surgery

Mice (10–14 weeks old) were injected intraperitoneally with a triple mixture of Medetomidine (0.75 mg/kg, Domitor; Nippon Zenyaku Kyogo Co., Ltd., Japan), Butorphanole (5 mg/kg, Meiji Seika Pharma Co., Ltd., Tokyo, Japan), and Midazolam (4 mg/kg, Fuji Pharma Co., Ltd., Japan). Following this, the mice were placed in a stereotactic apparatus (Narishige, Tokyo, Japan). Following the end of surgery, mice receive an intramuscular injection of 1.5 mg/kg atipamezole (Antisedan; Nippon Zenyaku Kyogo Co., Japan) which was given to antagonize Medetomidine to recover from sedation and Ringer’s solution (0.5 ml/mouse, i.p.; Otsuka, Japan) was injected. Mice were transferred back to the home-cage on an electric heated pad for 1 day then kept for 3 weeks to recover from operation before starting the behavior experiment. All virus injections were performed by using a 10 µl Hamilton syringe (80,030, Hamilton, USA) that was fitted with mineral oil-filled glass syringe separating the virus and wired to an automated motorized microinjector IMS-20 (Narishige, Japan). Injection started 5 min after inserting the capillary tube, which remained in place 10 min following termination of injection to ensure proper virus diffusion with injection rate (0.1 μl/min).

*For chemogenetics inhibition experiment*, 500 nl of AAV2/9- CaMKIIa-hM4Di-mCherry viral vector was injected bilaterally into the mPFC targeting the PrL coordinates [from bregma: +2 mm anteroposterior (AP), ±0.35 mm mediolateral (ML), +1.8 mm dorsoventral (DV) from skull surface].

### Auditory-gated patience-to-action task

*Arena setup* All the behavior experiments were done in a soundproof room in an L-shaped arena composed of two equally sized contexts connected by a central square junction. The waiting context was a gray square (220 mm length × 300 mm height) with a smooth white acrylic floor and walls without pattern. The open end of the waiting context was highlighted by a gray tape as a context boundary cue. The side context which includes the lickport is a transparent square context (220 mm length × 300 mm height) with a pointy texture green floor and walls with characteristic patterns (Black vertical lines on a white background). The two contexts (waiting context and side context) are connected together through a square junction without boundaries (220 mm length × 300 mm height) with a white acrylic floor and 2 walls. In the middle of one wall, a tape of light is hanging which turns on upon correct exit timing and turned off after licking. The wall facing the waiting context is used to hang the LED light strap vertically to be obvious for the mice during taking the decision to exit the context. The light visual cue has a behavioral significance on different levels, (1) Light on → It indicates a reward chance duartion so mice can associate the light with sucrose delivery from the lickport so it encourage mice to approach the lickport. “light on-reward association”. (2) Light on then light off → It indicates trial end and mice have to rapidly leave the lickport and initiate a new trial for efficient task execution. “light off-trial end association” (3) Light on or light off after auditory signals → It indicates as a feedback for mice exit timing. The LED is placed just facing the waiting context exit location so mice could flexibily and rapidly correct their behavior even before approaching the lickport. “light-exit time feedback”. The side context has a drilled circular hole on the back wall (50 mm height from the floor with a diameter 20 mm). Lick port is fitted in the hole of the side context which is used to deliver 5 μL after correct choice. Mice motion is tracked by a Video tracking simulator 4 (VTS-4) which initiates the trial upon entering the waiting context and tracks mice motion across the arena.

*Water restriction* Mice were kept under a water restriction protocol starting during the habituation phase and continuing through the whole task. Mice weight was kept at 80–85% of their original weight to keep their motivation for task execution.

*Handling* This phase starts 3 days before any habituation or behavior session. The purpose of this phase is to relief any stress occurrence for the mice during the exposure with the experimenter to avoid any difficulties or delay in task learning. Handling by using a cylindrical tunnel (mouse tunnel K3323, AS ONE Japan) and cupping method was used which has a positive impact to reduce mice stress compared to the tail hanging method. One day before handling starts, the tunnel was introduced to the mice home cage to be habituated to enter it. From first handling day, mice were picked up with the tunnel and then they started to move out to experimenters` hands. The same procedure was repeated for the 3 days until at the final day, mice were picked up directly to the hands without picking up the tunnel.

*Habituation* This phase lasts for 3 days including 3 habituation inputs: arena, tones and lick ports. Mice are habituated for the 3 days for the arena. On the first and second days, mice are habituated to each context individually by using a door for each one. In the 2nd day, mice are habituated to listening the tones in home cage to avoid fear from listening to them in behavior sessions. In the 3rd day, mice are habituated for the lick port licking and reward delivery in a separate home cage with a drilled hole for lick port fitting.

*Tones presentation* Tones are presented by a speaker (Elecom) that can produce a range of tone frequencies. Tones volume was maintained at ≈70 db. In every trial, tone signals were constructed by delivering 500 ms tone frequencies separated by 200 ms inter tone interval (ITI).

*Training* Days (7–10), a sequential representation of tone frequencies composed of initial frequency of 6 kHz as a start signal. After ITI, the same 6 kHz is repeated again which should represent hold cue. Then, after ITI a different tone frequency (12 kHz) is represented which acts as a go signal. For trial initiation, mice have to enter the waiting context for auditory signals representation. After the end of the 3 tones, mice are given a chance time of 5 s to exit the waiting context. Upon exiting at correct timing, LED light would turn on to give feedback of correct timing and permitting sucrose delivery upon poking a lick-port. Mice have to approach the lick port during the reward chance (5 s) to get a sucrose water reward. Mice can use the light signals as feedback for their action timing to promote the efficacy of their behavior. If mice did not exit the waiting context in chance time, this will lead to the start of a new trial and tone representation starts again and it will be counted as a missed exit trial. If mice missed approaching the lick port during the reward chance time, LED light will be turned off to indicate the end of the trial and it will be counted as a missed lick trial. If mice made an early exit before the end of the Go signal, the LED light would be kept off which indicates a premature exit timing, and no reward chance will be initiated. In all of these cases, mice have to return back to the waiting context to initiate a new successful trial to acquire a sucrose reward. Each session is fixed to 75 min time duration to allow comparing the number of rewards delivered in each session across days and between groups.

The arena was cleaned using water and 70% ethanol after each subject or session. Sleep deprivation was done by gentle shaking of the home cage after the end of the session for 4 h.

*For chemogenetic inhibition experiment* It was conducted in the same manner as previously described in the behavior establishment. On day 7, single CNO or 5% DMSO in saline (5 mg/kg) i.p injection was delivered to mice 30 min before the behavior session and then another injection to be administered after the behavior session. On day 8, single CNO or DMSO in saline (5 mg/kg) was administered 30 min before the behavior session.

### Behavioral analysis

All sessions were recorded by an overhead web camera (Logitech HD pro C920) mounted on a vertical stand. Bandicam screen recording was used to record the behavior with TDT circuit profile inputs. The parameter used to evaluate behavioral aspects in this task was calculated as follows:

$$\% Full successful behavior=\frac{Number of rewards}{Total number of trials} \times 100$$

### Immunohistochemistry c-fos staining

90 min after behavior session on day 8 or after water restriction on home cage, mice were deeply anesthetized using 0.5 ml of 3 drugs anesthesia mixture and transcardially perfused with phosphate buffer saline (PBS) (pH 7.4), followed by 4% formaldehyde in PBS (PFA) solution. Brains were carefully extracted from the skull and then fixed in PFA overnight at 4 °C. The subsequent day, brains were immersed in a 25% sucrose in PBS solution at 4 °C for 24 h before being carefully dried then, frozen in dry ice powder and stored at −80 °C. To get coronal sections, brains were cut into 50 µm sections using a cryostat (Leica CM3050, Leca Biosystems) and then allowed to rest in room temperature PBS solution in a 12-well cell culture plate (Corning, NY, USA). Sections were then placed in a blocking buffer consisting of 3% normal donkey serum for 1 h (S30, Chemicon by EMD Millipore, Billerica, MA, USA) in PBS containing 0.2% Triton X-100 and 0.05% Tween 20 (PBST) at room temperature. After the incubation time, sections were then washed three times with 10 min intervals with PBST before washing the buffer and then being incubated in a primary blocking solution with rat anti-c-Fos (1:1000, Synaptic systems, 226017). The 12-well plate carrying the sections was then wrapped in parafilm and aluminum foil and incubated at 4 °C for 36 h. At the end of incubation period of the 1ry antibody, sections were washed three times with PBST with 10 min interval before being incubated in a secondary blocking solution containing DAPI (1 μg/ml, Roche Diagnostics, 10236276001), together with donkey anti-rat IgG Alexa Fluor 488 (1:1000, Invitrogen, A21208). At the end, sections were washed three times with PBST with 10 min interval then last time with PBS before mounting onto glass slides using Immu-Mount (9990402, Thermo Fisher Scientific Inc.) with ProLong Gold anti fade reagent (Invitrogen).

### Histology

For the chemogenetics, following the behavior timeline end, mice were transcardially perfused, stored and sectioned as described in the previous section. For native nuclear staining, sliced sections were treated with a DAPI solution (1 μg/ml, Roche Diagnostics, 10236276001) and washed with PBS 3 times with 5 min interval then mounted on glass slides using Immu-Mount (9990402, Thermo Fisher Scientific Inc.).

### Confocal microscopy

Images were acquired using a confocal microscope (Zeiss LSM 900, Carl Zeiss, Jena, Germany) with 20× Plan-Apochromat objective lens. All parameters such as photomultiplier tube assignments, pinhole sizes and contrast values were standardized within each magnification and each experimental condition.

### Statistical analysis

Statistical analyses were carried out with GraphPad Prism 9 (GraphPad Software). An unpaired Student’s *t*-test was used to compare data between two different groups. ANOVA with post hoc tests, as detailed in each panel legend, was used for multiple-group comparisons. Quantitative data are reported as the mean ± s.e.m.

## Supplementary Information

Additional file 1.

Additional file 2.

## Data Availability

Data is provided within the supplementary information files.

## Abbreviations

AAV: Adeno-associated virus
AP: Anterior-posterior
CNO: Clozapine n-oxide
DAPI: 4,6 Diamidino-2-phenylindole
DREADD: Designer receptors exclusively activated by a designer drug
DMSO: Dimethyl sulfoxide
DV: Dorsal-ventral
ITI: Inter-tone interval
LED: Light emitting diode
ML: Medial-lateral
mPFC: Medial prefrontal cortex
PBS: Phosphate buffered saline
PFA: Paraformaldehyde
PrL: Prelimbic cortex
RM: Repeated measures
S.D: Sleep deprivation
SD: Standard deviation
VTS-4: Video tracking simulator 4
